# Selection of reference genes for quantitative real-time PCR analysis in halophytic plant *Rhizophora apiculata*

**DOI:** 10.7717/peerj.5226

**Published:** 2018-07-12

**Authors:** Ankush Ashok Saddhe, Manali Ramakant Malvankar, Kundan Kumar

**Affiliations:** Department of Biological Sciences, Birla Institute of Technology & Science Pilani, K K Birla Goa Campus, Zuarinagar, Goa, India

**Keywords:** Reference gene, *Rhizophora apiculata*, Quantitative RT-PCR, Mangrove, Salt stress

## Abstract

*Rhizophora apiculata* is a halophytic, small mangrove tree distributed along the coastal regions of the tropical and subtropical areas of the world. They are natural genetic reservoirs of salt adaptation genes and offer a unique system to explore adaptive mechanisms under salinity stress. However, there are no reliable studies available on selection and validation of reference genes for quantitative real-time polymerase chain reaction (qRT-PCR) in *R. apiculata* physiological tissues and in salt stress conditions. The selection of appropriate candidate reference gene for normalization of qRT-PCR data is a crucial step towards relative analysis of gene expression. In the current study, seven genes such as elongation factor 1α (*EF1*α), Ubiquitin (*UBQ*), β-tubulin (β-*TUB*), Actin (*ACT*), Ribulose1,5-bisphosphate carboxylase/oxygenase (*rbcL*), Glyceraldehyde 3-phosphate dehydrogenase (*GAPDH*), and 18S rRNA (18S) were selected and analyzed for their expression stability. Physiological tissues such as leaf, root, stem, and flower along with salt stress leaf samples were used for selection of candidate reference genes. The high-quality expression data was obtained from biological replicates and further analyzed using five different programs such as geNorm, NormFinder, BestKeeper, Delta Ct and RefFinder. All algorithms comprehensively ranked *EF1*α followed by *ACT* as the most stable candidate reference genes in *R. apiculata* physiological tissues. Moreover, β*-TUB* and 18S were ranked as moderately stable candidate reference genes, while GAPDH and *rbcL* were least stable reference genes. Under salt stress, *EF1*α was comprehensively recommended top-ranked candidate reference gene followed by *ACT* and 18S. In order to validate the identified most stable candidate reference genes, *EF*1α, *ACT*, 18S and *UBQ* were used for relative gene expression level of sodium/proton antiporter (*NHX*) gene under salt stress. The expression level of *NHX* varied according to the internal control which showed the importance of selection of appropriate reference gene. Taken together, this is the first ever systematic attempt of selection and validation of reference gene for qRT-PCR in *R. apiculata* physiological tissues and in salt stress. This study would promote gene expression profiling of salt stress tolerance related genes in *R. apiculata*.

## Introduction

Mangroves are a unique intertidal ecosystem and evolutionarily adapted to the interface between land and water environments (Saddhe, Jamdade & Kumar, 2017). They are distributed along the tropical and subtropical part of the world and consist of 73 mangrove species with few recognized hybrids in 123 countries covering of 150,000 km^2^ globally (Spalding, Kainuma & Collins, 2010). *Rhizophora apiculata* is a hardy woody fast growing mangrove tree. They are distributed throughout the Indian coastal region but the dominant population is on the southern coast of India (Menon & Soniya, 2014). They can tolerate salinity up to 65 parts per thousand (ppt) and show optimum growth at 8–15 ppt salinity (Robertson & Alongi, 1992). Mangrove plants are always exposed to the local hostile environments such as fluctuated water level, marshy land with anoxic conditions, hypersalinity and high UV light exposure (Tomlinson, 1986; Hutchings & Saenger, 1987). In order to survive in harsh conditions, they have developed some specialized traits such as viviparous propagules, aerial extensive supporting roots and high content of secondary metabolites. They are non-secretors and store surplus salt that enters through the transpiration stream into their leaves (Menon & Soniya, 2014). Mangroves are natural salt tolerant plant species but there are very few reports available on salt tolerance mechanism and salt stress associated genes. Several salt-induced genes were isolated and characterized from *R. apiculata* using suppression subtractive hybridization technique (Menon & Soniya, 2014). All salt-induced genes were highly upregulated at 12 h and further confirmed by qRT-PCR analysis using Actin (*ACT*) as a reference gene (Menon & Soniya, 2014). Recently *de novo* genome assembly of *R. apiculata* was reported (Xu et al., 2017), but the sequence is not accessible. Moreover, comparative transcriptome analysis was performed in mangroves species such as *Bruguiera gymnorrhiza, Kandelia obovata, R*. *apiculata*, and *Ceriops tagal* to understand adaptive evolution in the harsh intertidal habitats (Xu et al., 2017; Guo et al., 2017). However, there are no systematic reports available on selection and validation of reference gene for qRT-PCR in *R. apiculata* species.

Several techniques are available to investigate gene expression analysis including, semi-quantitative reverse transcription polymerase chain reaction, northern blotting, *in situ* hybridization, and quantitative real-time PCR (qRT-PCR). The qRT-PCR is a reliable, sensitive, and wide quantification range gene expression analysis technique (Bustin, 2002). Moreover, reference gene for qRT-PCR normalization is not universally standardized and it varies according to plant tissue material and experimental conditions (Bustin et al., 2009). For precise quantification and reproducible profiling, selection and validation of stable candidate reference genes are crucial steps prior to qRT-PCR for data normalization. Some commonly used reference genes include *Actin* (*ACT*)*,* β-*tubulin (TUB), Ubiquitin* (*UBQ*), *Glyceraldehyde 3-phosphate dehydrogenase* (*GAPDH*), elongation factor 1α (*EF1*α**) and 18S ribosomal RNA (18S) that are preferred to normalize the expression profiles of candidate genes. These reference genes are involved in basic cellular functions, maintaining cell size and shape, and cellular metabolism (Bustin, 2002). However, several reports have shown that the level of reference genes expression varies in different cultivars, tissues, and stress conditions (Sinha et al., 2015; Reddy et al., 2015; Nikalje et al., 2018). Hence, it is very important to select and validate most appropriate reference genes involved in various experimental conditions before proceeding to gene expression analysis. Various web-based tools and algorithms are available to address validation of candidate reference genes including, comparative ΔCt (cycle thresholds) (Silver et al., 2006), NormFinder (Andersen, Jensen & Orntoft, 2004), BestKeeper (Pfaffl et al., 2004), and geNorm algorithm (Vandesompele et al., 2002). RefFinder, a web-based program, which provides a comprehensive ranking of reference genes (Xie et al., 2012).

Based on the literature survey, there were no reports available on evaluation of candidate reference genes for qRT-PCR in *R. apiculata*. In the present study, we aim to evaluate the most stable candidate reference gene for qRT-PCR gene expression analysis in *R. apiculata* physiological tissues and in salt-stressed leaf samples. The current study will promote the gene expression analysis in the *R. apiculata,* especially when studied under salinity stress.

## Materials & Methods

### Plant materials

In the present study, we collected three month old *R. apiculata* seedlings located in the west coast of India with the geographical latitude of 15.5256°N and longitude of 73.8753°E, with the permission from the Principal Chief Conservator of Forest, Goa Forest Department, Goa, India. Mangrove species identification was performed based on morphological characteristics using a comparative guide to the mangroves of Goa (Naskar & Mandal, 1999). All seedlings were acclimatized and maintained in half-strength Hoagland solution at a temperature regime of 24–30 °C, 40–50% relative humidity. Various physiological tissues such as leaves, stems, roots and flower samples were collected. To imitate salt stress conditions, young seedlings of *R. apiculata* were exposed to Hoagland nutrient solution supplemented with 250 mM sodium chloride (NaCl) continuously and leaf samples were harvested at different time-course such as 0, 6, 12 and 24 h.

### RNA isolation and cDNA synthesis

Total RNA was extracted using modified cetyl-trimethyl ammonium bromide (CTAB) protocol with 2% polyvinylpyrrolidone and 10% β-mercaptoethanol (Fu et al., 2004). Freshly collected tissues were immediately pulverized into 2 ml of pre-warmed CTAB buffer and incubated at 60 °C for 30 min. The suspension was gently mixed and centrifuged at 14,000 rpm for 10 min at room temperature with an equal volume of chloroform: isoamyl alcohol (24:1). The aqueous phase was transferred to a new tube and RNA was precipitated with a 1/3^rd^ volume of 8M lithium chloride (LiCl) and incubated at −20 °C for 1 h followed by adding an equal volume of chilled isopropanol (−20 °C). The RNA was precipitated by centrifugation at 14,000 rpm for 10 min at room temperature followed by washing with 70% ethanol. RNA was finally dissolved in 0.1% DEPC treated water and its quantity and quality were confirmed by Nanodrop (Thermo Fisher Scientific, Waltham, MA, USA). Genomic DNA contamination was removed by DNase I enzyme (Thermo Fisher Scientific, Waltham, MA, USA) treatment at 37 °C for 30 min and heat inactivated at 65 °C for 10 min with 50 mM EDTA. The cDNA synthesis was performed in 20 µl reaction volume using the RevertAid Reverse Transcriptase (Thermo Fisher Scientific, Watham, MA, USA), 0.1–5 µg RNA sample and oligo d(T)_18_ primer, as per manufacturer’s instructions.

### Selection of reference genes and primer designing

Nine housekeeping genes such as *ACT,*α*-TUB,* β*-TUB*, *GAPDH, UBQ*, 18S rRNA, *rbcL*, Histone H3 and *EF1*α** used in qRT-PCR along with one target gene sodium/proton antiporter (*NHX*) were selected. There is no genome sequence available publicly for *R. apiculata* hence, homologous candidate reference gene sequences were retrieved from model plants such as *Arabidopsis thaliana* and *Oryza sativa* from Gramene and NCBI databases. Full-length candidate reference gene sequences were used for primer designing using PrimerQuest (Integrated DNA Technologies) with given parameters: melting temperature (T_m_) of 55–65 °C, primer length of 17–25 bp, and amplicon length of 100–500 bp (Table 1). The amplicon was sequenced and annotated based on the sequence similarity-based search tool. Further, all the confirmed sequences were submitted to GenBank for accession numbers. After primer specificity analysis α-*Tub* and Histone H3 were removed from further analysis. The primer sequences, accession numbers, and their efficiency were given in Table 1.

**Table 1:** Details of candidate reference genes, Accession number, primer sequences, amplicon size, PCR efficiency (%) and regression coefficient (*R*^2^) for each candidate reference gene selected in this study.

Sr. no.	Gene label	Accession No.	Gene description	Primer sequence (5′-3′)	Amplicon size (bp)	PCR efficiency (%)	*R*^2^
1	18S	MH277331	18S rRNA	F-CCGTCCTAGTCTCAACCATAAAC R-GCTCTCAGTCTGTCAATCCTTG	189	102.30%	1
2	*ACT*	MH279969	Actin	F-ATCACACCTTCTACAACGAGC R-CAGAGTCCAACACGATACCAG	207	92.03%	0.994
3	*EF1*α**	MH310424	Elongation Factor 1 α	F-AGCGTGTGATTGAGAGGTTC R-AGATACCAGCCTCAAAACCAC	53	98.60%	0.99
4	*UBQ*	MH310425	Ubiquitin	F-CACTTCGACCGCCACTAC R-AGGGCATCACAATCTTCACAG	60	90.54%	0.992
5	*RbcL*	KP697362	Ribulose 1,5-Bisphosphate Oxygenase/Carboxylase Large	F-ATGTCACCACAAACAGAGACTAAAGC R-GTAAAATCAAGTCCACCRCG	530	97.69%	0.996
6	β*-TUB*	MH310423	β-tubulin	F-ACCTCCATCCAGGAGATGTT R-GTGAACTCCATCTCGTCCATTC	60	94.08%	0.996
7	*GAPDH*	MH279970	Glyceraldehyde3-phosphate dehydrogenase	F-ACCACAGTCCATGCCATCAC R-TCCACCACCCTGTTGCTGTA	264	96.78%	0.99
8	*NHX*	KU525079	Sodium/proton antiporter	F-TGCTAGCTCTTGTCCTGATTG R-ATTGACACAGCACCTCTCATTA	120	103.70%	0.997

For qRT-PCR, primer specificity was determined using melting curve analysis and the PCR products were checked on 2% agarose gel. The primer efficiency of all candidate reference genes was calculated based on the standard curve generated from a 10-fold serial dilution of cDNA (10^0^, 10^−1^, 10^−2^, and 10^−3^) and regression coefficient (*R*^2^) values. Primer efficiency was calculated using the given formula [*E* = (10^(−1∕slope)^ − 1) × 100], where *E* = 2 and corresponds to 100% efficiency; high/acceptable amplification efficiency equals 90–110% (Sinha et al., 2015).

### Quantitative RT-PCR analysis

The quantitative RT-PCR analysis was carried out using SYBR green master mix (2X Brilliant III SYBR^^®^^ Green QPCR; Agilent Technologies, Santa Clara, CA, USA), on AriaMx Agilent system (AriaMx; Agilent Technologies, Santa Clara, CA, USA) with the following reaction conditions: initial denaturation at 95 °C for 3 min, 40 cycles of 95 °C for 30 s, 55 °C for 30 s and 72 °C for 45 s extension, and a melt-curve program (65–95 °C with a temperature increase of 0.5 °C after every 5 s). The melting curve was generated to determine the amplicon specificity. The qRT-PCR experiments were performed using three biological and two technical replicates. A reaction with no template control and a reverse transcription negative control were performed to check the potential reagents and genomic DNA contamination.

### Analysis of gene expression stability

The candidate reference gene ranking was analyzed using five different algorithms such as geNorm, NormFinder, Bestkeeper, Δct, and comprehensive ranking analysis by RefFinder.

### geNorm analysis

The geNorm determines the most stable reference genes based on the gene expression stability value (*M*) for a reference gene. It also calculates the minimum number of candidate reference genes required for normalization of target genes. It requires calculated Cq values into relative quantities using the given formula: *Q* = *E*^(minCq−Cq)^, where Q represents sample quantity relative to sample with the highest expression, E is amplification efficiency and min Cq is the lowest Cq values. The stability value (*M*) is defined as an average pairwise variation (V) of the gene compared with all other tested reference genes and the cut-off is 1.5 (Vandesompele et al., 2002). If *M* value is lower than 1.5, it represents stable candidate reference gene and higher values reflect least stable.

### NormFinder

NormFinder calculates expression stability values for candidate reference genes and evaluates the most stable reference gene pairs. It also calculates intra and intergroup variation using a direct comparison between genes. It uses same input calculation files which are required for geNorm with a little variation such as the first row represents a sample, the first column represents genes and the last row represents a group of samples. NormFinder is available with Excel spreadsheet add-in (https://moma.dk/normfinder-software). It ranks candidate reference genes based on expression stability value. Lowest *M* value represents most stable reference gene and higher the value, least stable are the genes (Andersen, Jensen & Orntoft, 2004).

### BestKeeper analysis

BestKeeper determines the best reference gene based on the normalization factor (also called Bestkeeper index) and pairwise correlation analysis. It requires raw Cq values as an input data to select most stable and least stable candidate reference genes. It is available in an MS Excel spreadsheet file (http://www.gene-quantification.de/bestkeeper.html) and in RefFinder (http://150.216.56.64/referencegene.php?type=reference) as well. It evaluates the candidate reference gene stability by comparing the standard deviation of each gene and averages of these values. It also calculates the coefficient of variance, Pearson correlation coefficient (*r*) values, geometric mean (GM) and arithmetic mean (AM).

### ΔCt method

This tool is available in an MS Excel spreadsheet as well as in RefFinder (http://150.216.56.64/referencegene.php?type=reference). It calculates the stable candidate reference gene based on standard deviation and pairwise comparison with other genes. ΔCt requires raw Cq values as an input data. It considers a pair of gene for calculations and compares ΔCt values among genes (Silver et al., 2006).

### RefFinder analysis

RefFinder is a web-based comprehensive tool developed for evaluating and screening reference genes from extensive experimental datasets. RefFinder was used to generate comprehensive stability rankings (Xie et al., 2012). Comprehensive ranking of seven candidate reference genes was analyzed using RefFinder.

### Validation of candidate reference genes

The reliability of highly stable candidate reference genes identified in the current study was validated using sodium/proton antiporter (NHX) as a salt stress target gene. The differential gene expression profiles of NHX under salt stress at 0, 6, 12 and 24 h were normalized using *EF1*α*, ACT*, 18S and *UBQ* along with the combination of *EF1*α + *ACT* genes. The input values for *EF1*α + *ACT* were calculated using the geometric mean formula given below to normalize gene of interest Geometric $Mean=\sqrt[n]{\times 1,}\times 2,\times 3\ldots .\times n$, where the *n* = number of times (Vandesompele et al., 2002). The average Cq values from three biological replicates were used for relative expression analysis and the relative gene expression level calculated using the 2^−ΔΔ^CT^^ method (Livak & Schmittgen, 2001; Manuka, Saddhe & Kumar, 2018). Statistical analysis was performed using SPSS 15.0 for Windows evaluation version to verify the significant difference between relative gene expressions. One-way Analysis of variance (ANOVA) with Tukey’s Honest Significant Difference (HSD) test was performed for comparison between reference genes and target genes. A *p*-value <0.05 was considered statistically significant.

### Minimum Information for publication of qRT-PCR experiments guidelines (MIQE)

All the qRT-PCR experiments and data analysis in the present study were performed in accordance with the MIQE guidelines (Bustin, 2002).

## Results

### Expression profiling of selected reference genes

**Figure 1:**
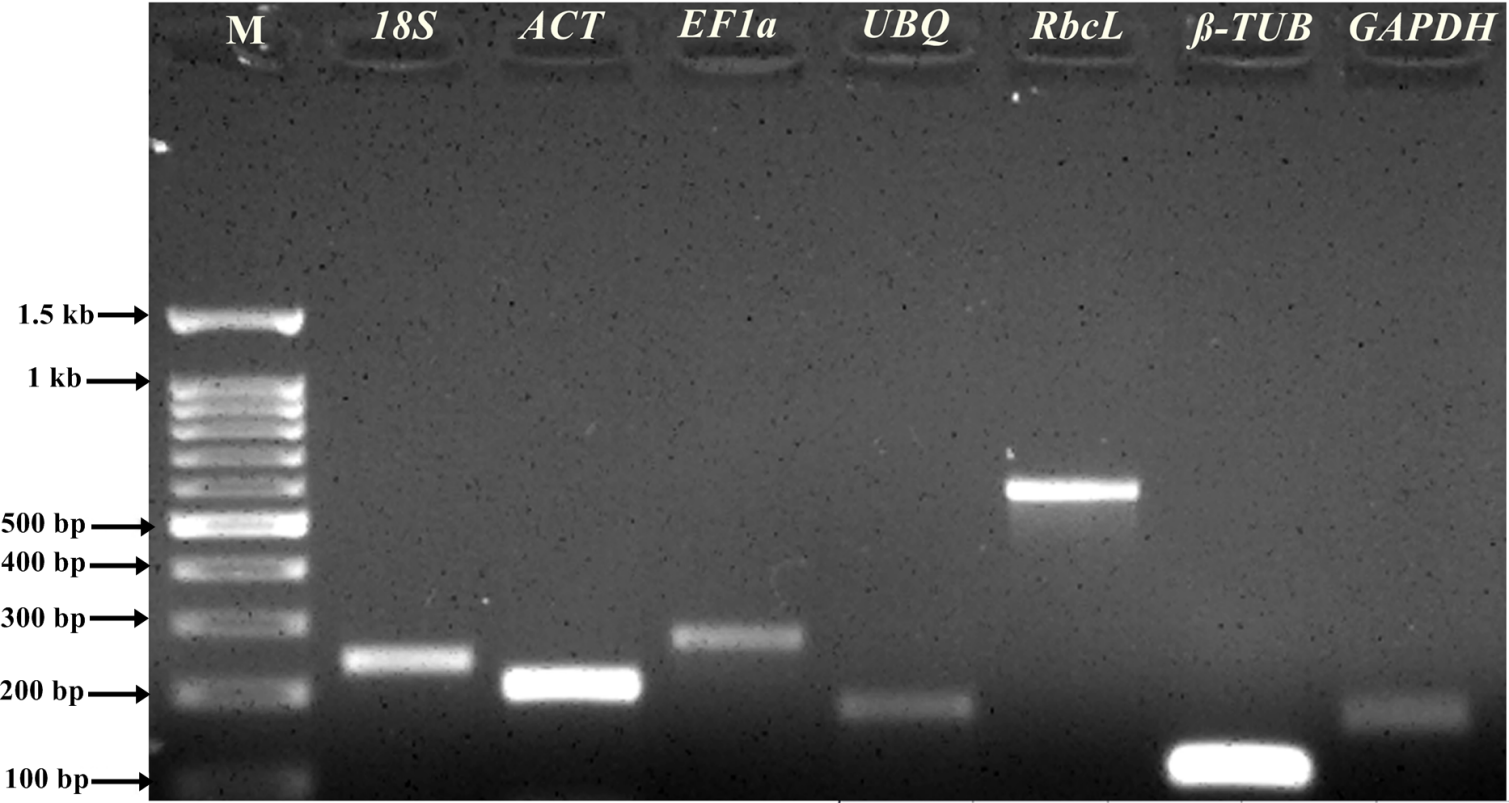
Amplification product of genes. PCR products on 2% agarose gel stained with ethidium bromide. Amplification products of seven candidate reference genes selected for gene validation of *R. apiculata* samples. M: 100 bp DNA ladder. Lanes 1, 2, 3, 4, 5, 6 and 7 were the gene products of 18S, *ACT*, *EF1*α**, *UBQ*, *RbcL*, ** β*-TUB,* and *GAPDH*, respectively.

**Figure 2:**
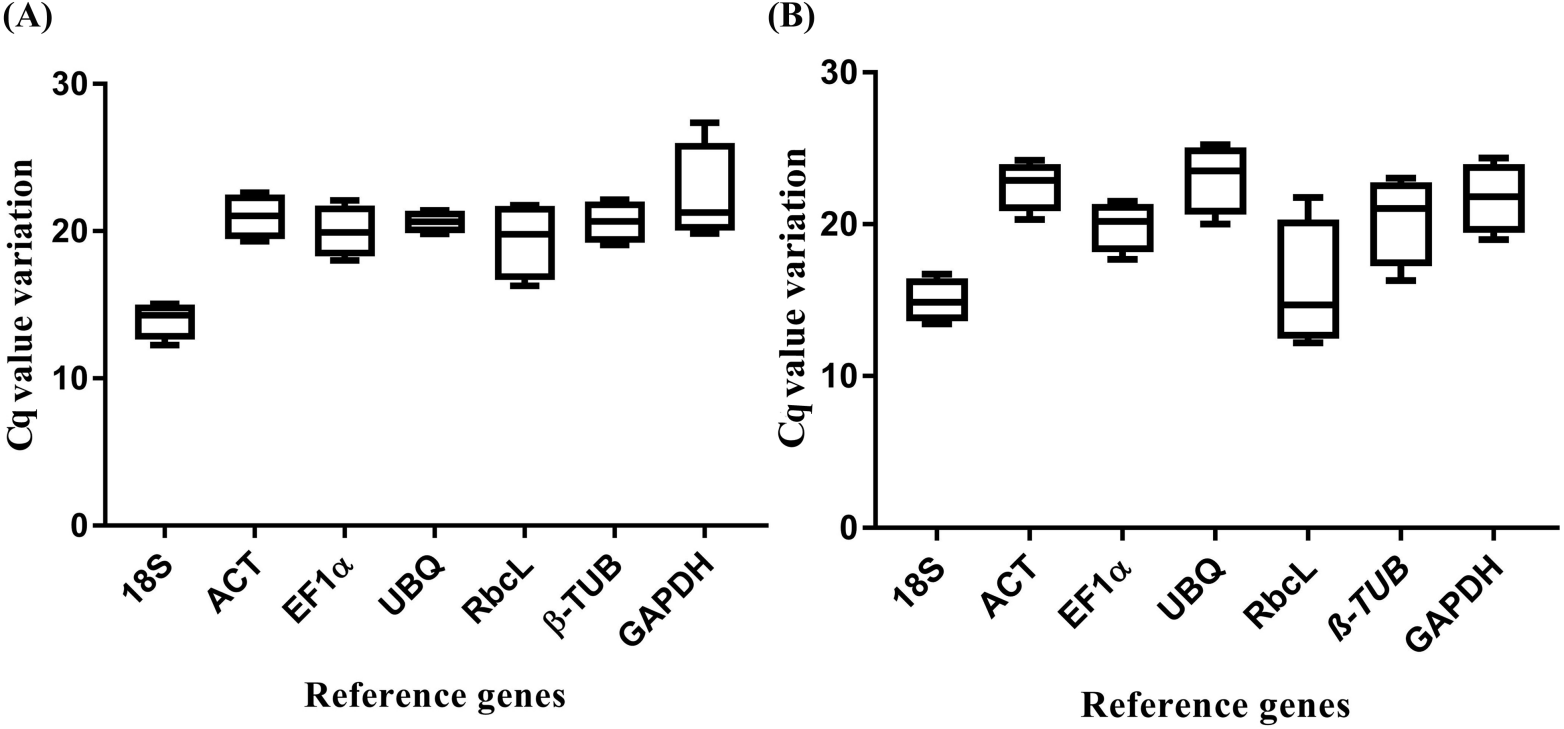
Threshold cycle (Ctq) values of seven candidate reference genes. (A) Tissue-specific box plot for the Cq values of seven candidate reference genes from the qRT-PCR analysis. For each reference gene, the line inside the box is the median. The top and bottom lines of the box are the first and third quartiles, respectively. The top and bottom whiskers represent the 5th and 95th percentiles. (B) Salt stress box plot for the Cq values of seven candidate reference genes from the qRT-PCR analysis. For each reference gene, the line inside the box is the median. The top and bottom lines of the box are the first and third quartiles, respectively. The top and bottom whiskers are the 5th and 95th percentiles, respectively.

In order to select stable reference genes, transcript levels in tissues such as leaf, root, stem, and flower as well as salt stress samples were quantified based on their cDNA concentration. The primer specificity was determined by PCR products wherein single, expected amplicon size was obtained (Fig. 1). The qRT-PCR melting curve for template test and negative control (NTC) without template were analyzed for primer-dimer and reagents contamination (Fig. S1). Further, NTC samples were confirmed by running 2% agarose gel electrophoresis. The amplified PCR products were sequenced and submitted to GenBank for accession numbers. All the sequenced PCR products were identified and annotated based on BLAST search. The primer efficiency (%) ranged from 103.70%, (*R*^2^ = 0.997) for *NHX* to 90.54% (*R*^2^ = 0.98) for *UBQ* including 18S (102.30, *R*^2^ = 1), *ACT* (92.03%, *R*^2^ = 0.994), *EF1*α** (98.60%, *R*^2^ = 0.99), β*-TUB* (94.08%, *R*^2^ = 0.996), *GAPDH* (96.78%, *R*^2^ = 0.992) and *RbcL* (97.69%, *R*^2^ = 0.996) (Fig. S2; Table 1).The mean cycle threshold (Cq) values of the seven selected reference genes for different tissue samples ranged from 14.16 for18S to 21.77 for *GAPDH* (Fig. 2A). Similarly, for the salt stress samples, the mean Cq values ranged from 13.96 for 18*S* to 24.23 for *UBQ* (Fig. 2B). Mean Cq values gave insight into approximate gene expression data. Negative control showed higher Cq values indicating no product amplification which was further checked on a 2% agarose gel. Moreover, negative control without reverse transcriptase did not show any product amplification, thus indicating no gDNA contamination.

### geNorm analysis

**Figure 3:**
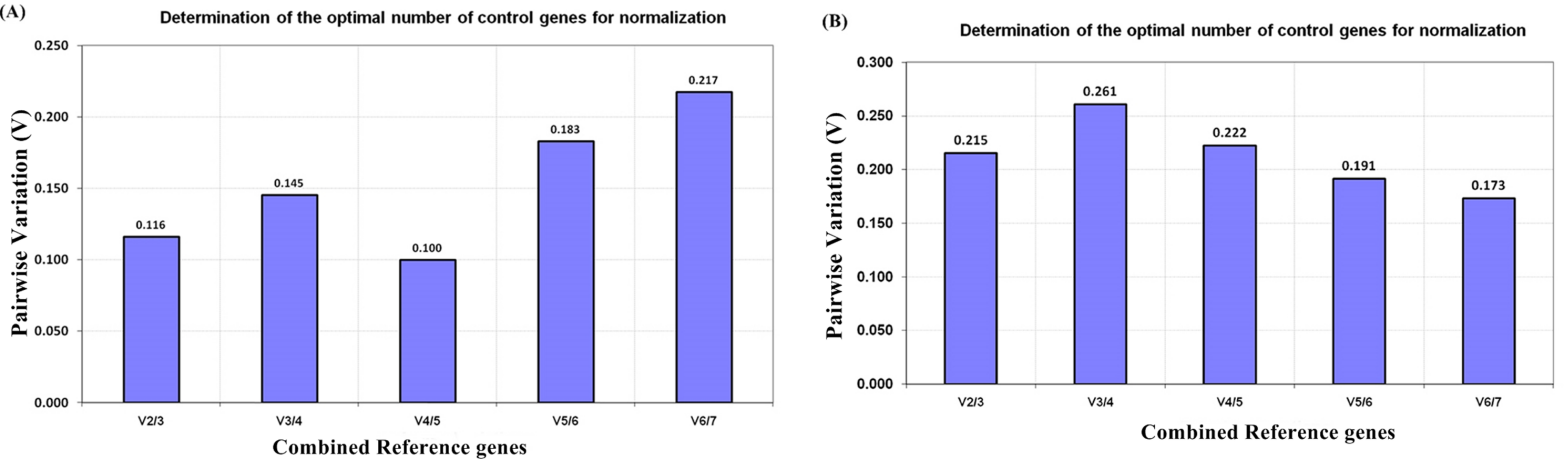
geNorm pairwise variation (V) analysis to determine minimum number of candidate reference genes required for normalization in qRT-PCR of *R. apiculata* (A) Pairwise variation analysis for physiological tissue samples (B) Pairwise variation analyzed for salt stress leaf samples. V1 to V7 stand for the variation in candidate reference genes ranked based on their stability, where V1 is the variation for the most stable and V7 is the variation for the least stable gene.

For physiological tissues, seven candidate reference genes showed average expression stability value (*M*) less than 1.5. *ACT* (*M* = 0.721) was most stable reference gene followed by *EF*1*α* (*M* = 0.761), and β*-TUB* (*M* = 0.763) (Table 2A; Fig. S3A). *GAPDH* was the least stable candidate reference gene with *M* value 1.599. The geNorm also determines an optimum number of candidate reference gene for normalization based on the calculation of pairwise variation (*V*_n_∕*V*_n+1_) between sequential normalization factor (*NF*_n_ and *NF*_n+1_). To select the best pair for normalization, the threshold value is 0.15. If pairwise variation value is lower than 0.15, there is no need to add more candidate reference gene. Moreover, the best pairwise variation value 0.382 was observed for a combination of *ACT* and *EF1*α and comprehensively recommended for normalization (Table 2B; Fig. S3A). Based on the observation, there were no effects on an addition of the third gene in the combination of *ACT* and *EF1*α which showed pairwise variation value below 0.15 (Fig. 3A).

**Table 2:** geNorm analysis and ranking of candidate reference genes based on stability value (*M*). Lower *M* value represents most stable reference genes and higher *M* value showed least stable reference genes.

A. geNorm analysis for individual candidate reference genes					
Sr. No	Reference genes	Physiological tissue samples		Salt stress samples	
		Stability value (*M* < 1.5)	Ranking	Stability value (*M* < 1.5)	Ranking
1	*18S*	0.880	4	1.020	2
2	*ACT*	0.721	1	0.927	1
3	*EF1*α	0.761	2	1.021	3
4	*UBQ*	0.928	5	1.399	7
5	*RbcL*	1.225	6	1.357	6
6	*B-TUB*	0.763	3	1.351	5
7	*GAPDH*	1.599	7	1.257	4

B. Best pair of reference genes based on geNorm analysis			
1	*ACT*+*EF1*α	0.382	Most stable pair of reference genes in physiological tissue samples
2	*ACT*+*EF1*α	0.462	Most stable pair of reference genes under salt stress samples

Under salinity stress, *ACT* was most stable candidate reference gene with *M* value 0.927, followed by 18S and *EF1*α showing same stability *M* value 1.02 (Table 2A). Moreover, *rbcL* and *UBQ* performed least stable candidate reference gene with *M* value 1.357 and 1.399 respectively. In salt stress, *ACT* + *EF1*α were the most suitable combination for normalization of the gene of interest with pairwise variation value of 0.462 (Table 2B; Fig. S3B). According to pairwise variation analysis, if the third gene was added in the *ACT* + *EF1*α*,* it showed higher pairwise variation value of 0.215 (Fig. 3B).

### NormFinder

In *R. apiculata* physiological tissue samples *EF1*α was most stable with stability value of 0.085. β*-TUB* (0.135) was the second most stable candidate reference gene followed by *ACT* (0.164) (Table 3A). *EF1*α and β*-TUB* (0.070) showed the most stable combination for the pair of candidate genes for normalization (Table 3B). Overall, *GAPDH, UBQ,* and *RbcL* were least stable reference genes. In salt stress, *ACT* was most stable reference gene with a stability value of 0.196. *EF1*α and 18S were second and third most stable candidate reference genes with stability value 0.257 and 0.273 respectively. *ACT* and *EF1*α showed the best pair of reference genes with stability value 0.183 (Table 3B). Under salt stress, geNorm and Normfinder showed almost similar results for a selection of candidate reference gene.

**Table 3:** NormFinder analysis and ranking of candidate reference genes based on stability value. Lower stability value represents most stable reference genes and higher value showed least stable reference genes. Ra-*Rhizophora apiculata*.

A. NormFinder analysis for individual candidate reference genes					
Sr. No	Reference genes	Physiological Tissue samples		Salt stress samples	
		Stability value	Ranking	Stability value	Ranking
1	18S	0.410	4	0.273	3
2	*ACT*	0.164	3	0.196	1
3	*EF1*α	0.085	1	0.257	2
4	*UBQ*	0.463	5	0.518	6
5	*RbcL*	0.500	6	0.499	5
6	*B*-*TUB*	0.135	2	0.533	7
7	*GAPDH*	0.568	7	0.483	4

B. Best pair of candidate reference genes based on geNorm analysis			
Sr. No.	Best pair of genes	Stability value	
1	*EF1α* + *B*-*TUB*	0.070	Most stable pair of candidate reference genes in Ra tissue samples
2	*ACT*+*EF1*α	0.183	Most stable pair of candidate reference genes in salt stress samples

### BestKeeper

In the BestKeeper analysis, standard deviation (SD) and coefficient of correlation (*r*) value were the criteria used for comparison. Highest *r* value represents the most stable candidate reference genes and lower *r* value represents the least stable genes. Here, we considered *r* value for evaluation, showing *EF1*α as the most stable reference gene followed by *ACT* with *r* value 0.987 and 0.966 respectively. *GAPDH* was ranked as the least stable candidate reference gene with lower *r* value (Table 4). The result is consistent with geNorm and NormFinder analysis. In salt stress, *ACT* (*r* = 0.638) showed most stability followed by β*-TUB* (*r* = 0.625) and *EF1*α (*r* = 0.523) (Table 4). Under salt stress, similar results were observed with little variation in BestKeeper. BestKeeper determined *β-TUB* second most stable candidate reference gene in salt stress.

**Table 4:** Candidates reference gene stability and ranking analyzed by BestKeeper (Coefficient of correlation, r), Ct (Mean, STDEV) ranking of genes. Coeff. of corr, Coefficient of correlation; RG, reference gene.

Sr. No	RG	BestKeeper				ΔCt analysis			
		Physiological Tissue samples		Salt stress samples		Physiological Tissue samples		Salt stress samples	
		Coeff. of corr. (r)	Rank	Coeff. of corr. (r)	Rank	Mean SD	Rank	Mean SD	Rank
1	*18S*	0.935	4	0.507	4	0.88	3	1.02	2
2	*ACT*	0.966	2	0.638	1	0.76	2	0.93	1
3	*EF1*α	0.987	1	0.523	3	0.72	1	1.02	2
4	*UBQ*	0.964	3	0.001	7	0.93	4	1.40	6
5	*RbcL*	0.958	5	0.310	6	1.22	5	1.36	5
6	*B*-*TUB*	0.964	3	0.625	2	0.76	2	1.35	4
7	*GAPDH*	0.850	6	0.435	5	1.60	6	1.26	3

### ΔCt analysis

According to ΔCt analysis*, EF1*α** was the most stable candidate reference gene followed by *ACT* and β*-TUB* in physiological tissue (Table 4)*.* 18S was ranked as an average or moderately stable reference gene. The results were consistent with earlier analysis. *GAPDH, RbcL,* and *UBQ* were the least stable. Under salt stress, *ACT* was most stable candidate reference gene followed by *EF1*α** and18S (Table 4).

### Comprehensive ranking of candidate reference genes

**Figure 4:**
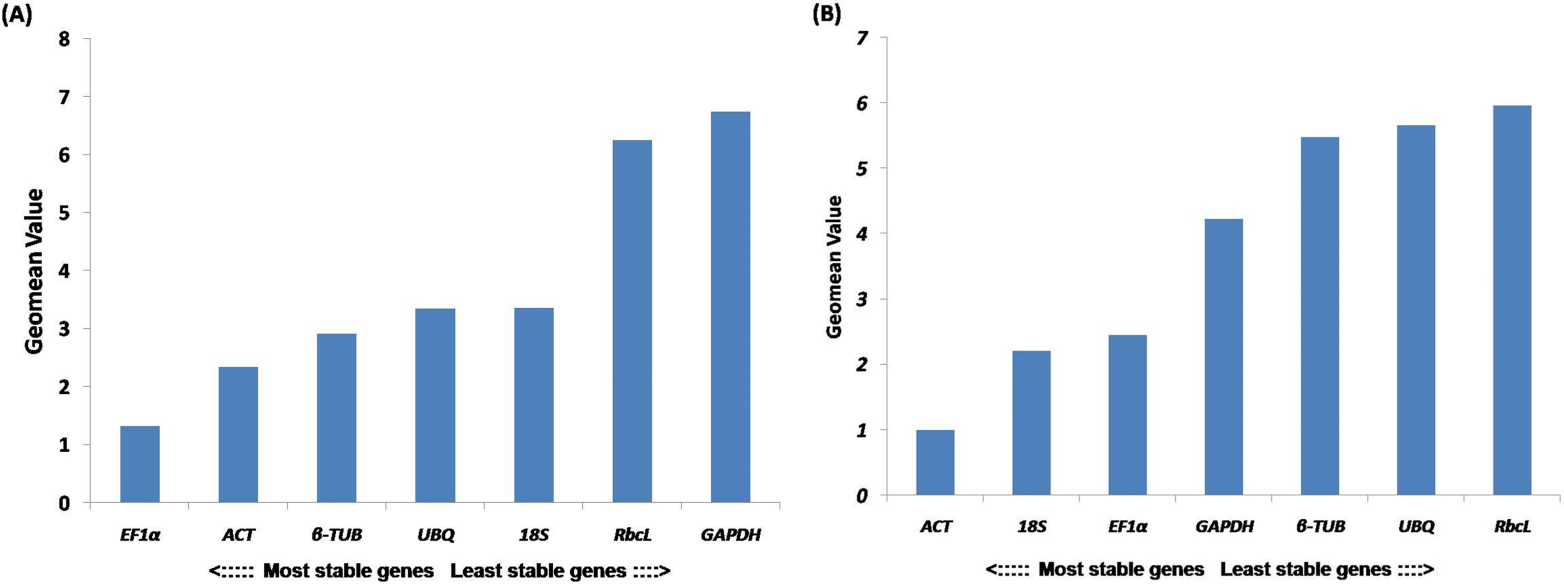
Ranking of reference genes. Comprehensive ranking of candidate reference genes in *R. apiculata* based on the rankings from each algorithms using RefFinder (A) Overall ranking of candidate reference gene in physiological tissues (B) Overall ranking of candidate reference gene in salt stress leaf samples.

Based on the Geomean value, a comprehensive ranking of all candidate reference genes showed *EF1*α** (1.32) was the most stable followed by *ACT* (2.34) and β*-TUB* (2.91) (Fig. 4A). Moreover, *rbcL* and *GAPDH* performed as a least stable candidate reference genes. In salt stress, *ACT* was the most stable with Geomean value 1. Moreover, 18S was second most stable candidate reference gene with Geomean value 2.29 (Fig. 4B). Here, *UBQ* and *rbcL* performed as least stable candidate reference genes.

### Validation of stable candidate reference genes under salt stress

**Figure 5:**
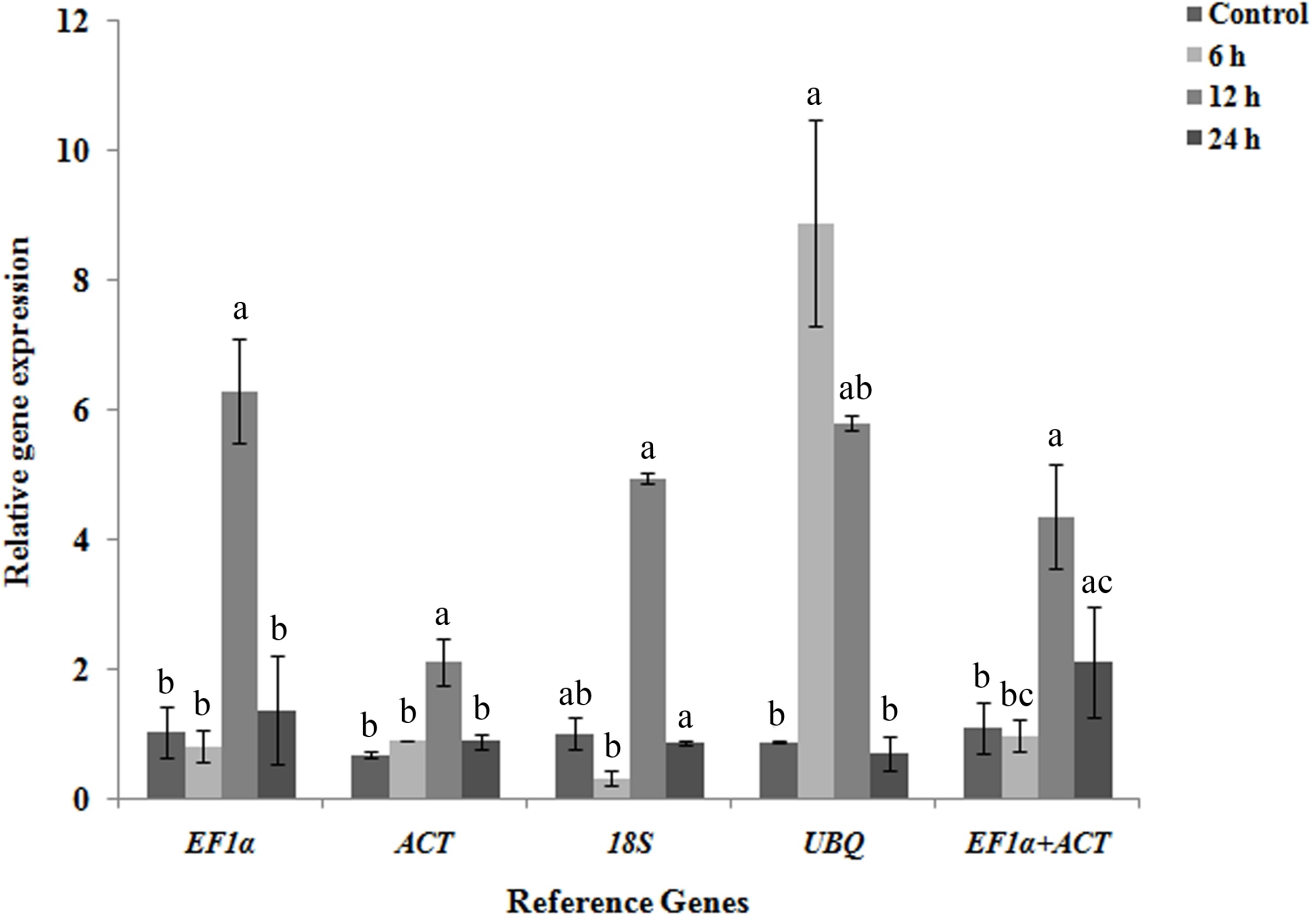
Validation and normalization of target NHX gene of *R. apiculata* under salt stress at four different time-course such as 0, 6, 12 and 24 h using EF1 α, ACT, 18S and *UBQ* reference genes. Normalization of NHX using *EF1 α, ACT,* 18S*, UBQ* and combined *EF1α* +* ACT*. Error bars represent the mean ± standard error of relative abundance of three biological replicates. The bars having different superscript letter are significantly different at *p* < 0.05.

To validate the efficacy of candidate reference genes, *ACT*, *EF1*α**, 18S and *UBQ* were used to normalize the expression levels of NHX in salt stress at four different time course. Set of the most stable candidate reference genes such as *ACT*, *EF1*α**, 18S and the least stable candidate reference gene *UBQ* were used as internal controls. While using EF1α, *ACT,* and 18S alone for normalization, *NHX* showed significant upregulation expression pattern in salt stress at 12 h. However, with *UBQ* as an internal control, NHX expression was upregulated in salt stress after 6 h of salt stress (Fig. 5).

## Discussion

The present study is the first systematic assessment of candidate reference gene in *R. apiculata* physiological tissues as well as under salt stress. The MIQE guidelines gives a framework for good experimental practice and transparent results (Bustin et al., 2009). The results were in accordance with the MIQE guidelines, where the ideal PCR efficiency is 100%, while the acceptable range is from 90 to 110% (Bustin et al., 2009). In the present study, we designed the primers based on homologous genes of *Arabidopsis thaliana* because genome sequence of *R. apiculata* is not available. To check the designed primers specificity, we tested PCR and confirmed on 2% agarose gel for single desired size bands. Further, amplified PCR products were confirmed through sequencing and identified by BLASTN tool. All the sequences were submitted to GenBank for accession numbers. In the present work, the primer efficiency ranged from 90–103% and most of the study reported primer efficiency ranging from 90–110%. The primer efficiency was recorded between 92 to 98.6% in *Sesuvium portulacastrum*, 92.89–98.76% in *Suaeda aralocaspica*, 81–100.88% in *Halostachys caspica*, 90.5–104.43% in *Cajanus cajan* (Nikalje et al., 2018; Cao, Wang & Lan, 2016; Zhang et al., 2016; Sinha et al., 2015).

Selection of unstable reference gene can lead to fallacious relative gene expression result and errors in normalization (Dheda et al., 2005). Besides the selection of suitable genes, it is equally important to select more than one candidate reference gene which improves the gene expression analysis (Vandesompele et al., 2002). The geNorm algorithm evaluates single as well as best pair stable candidate reference genes for normalization. In the current study, a comprehensive ranking of candidate reference genes was evaluated; *EF1*α** being the most stable candidate reference gene in physiological tissues and *ACT* in salt stress. The geNorm algorithm gave a consistent result with a comprehensive ranking which showed the most stable candidate reference gene as *EF1*α** in physiological tissues and *ACT* in salt stress tissue samples. A similar observation was reported in the *Halostachys caspica* halophyte species, which showed that *EF1*α** and *TUB3* was the most stable under salt and drought stress (Zhang et al., 2016). Under salt stress, most stable reference genes in *S. portulacastrum* shoot tissue were α*-TUB*, *EIF4a* and *EF1*α, while *UCE 2*, *TBP* and *EF1*α in the root tissue (Nikalje et al., 2018). This result reflects that a reference gene is not universal and altered according to plant species and stress conditions. So it is always recommended to select and validate the commonly used candidate reference genes. One of the possible reasons might include the differential expression patterns under unstressed and stressed conditions and a difference in response to the particular stress.

We observed a little variation in assessed best pair candidate reference genes between geNorm (*EF1*α +*ACT)* and NormFinder (*EF1*α + β*-TUB*) analysis. The possible explanation is subtle differences between their algorithm methods. Similar results were observed in earlier studies during evaluation of candidate reference genes, wherein a little variation in geNorm and NormFinder was reported, which leads to minute variation in candidate reference gene ranking, as reflected in the current study (Cruz et al., 2009; Pellino et al., 2011).

The geNorm calculates candidate reference genes to normalize target gene based on their average stability value (*M*) and also determines the optimum number of candidate reference genes required for normalization. Although NormFinder calculates stability values for each gene and BestKeeper ranks the genes according to *r* values, these algorithms do not determine the minimum number of reference genes required for normalization (Kozera & Rapacz, 2013). We have performed target gene validation using geNorm analyzed data because it ranks candidate reference genes based on their stability and also evaluates the minimum number of reference genes required for normalization. We used individual candidate genes as well as a combination of *EF1*α +*ACT*. We found that *EF1*α*, ACT,* and18S had given significant upregulation of *NHX* gene*,* while using least stable candidate reference gene *UBQ* showed different expression pattern after normalization. We observed that relative gene expression of NHX showed significant transcript accumulation pattern at 12 h. It was earlier reported that most significant expression patterns were observed in *R. apiculata* after 12 h time-course (Menon & Soniya, 2014). The geNorm data suggests the use of two reference genes for normalization of gene of interest. Moreover, most of the previous study underscored the use of more than one reference gene to improve the relative gene expression (Bustin, 2002).

In summary, we have successfully evaluated and validated stable reference genes in *R. apiculata* physiological tissues and under salt stress. This analysis revealed that the suitable reference genes differ between physiological tissues and in salt stress tissues. We found that commonly used reference genes such as *EF1*α** and *ACT* are most useful reference in an individual as well as in combined form.

## Conclusion

The current study examined the most stable candidate reference gene for the normalization of relative gene expression in *R apiculata* physiological tissue and under salt stress. We strongly recommend *EF1*α** followed by *ACT* and β*-TUB* as the best stable candidate reference genes for normalization in *R. apiculata* physiological tissue gene expression analysis. Under salt stress, *EF1*α** followed by *ACT* and 18S are the most suitable candidate reference genes for normalization. In conclusion, *EF1*α** and *ACT* can be used as candidate reference genes for the study of *R. apiculata*.

##  Supplemental Information

10.7717/peerj.5226/supp-1 Figure S1 Melting curve analysis qRT-PCR template and negative control (NTC) melting curve. Template melting curve (A) 18S, (B) *ACT*, (C) *EF1*α**, (D) *UBQ*, (E) *RbcL*, (F) ** β*-TUB* (G) *GAPDH* and (H) *NHX*. Negative control samples without template (NTC) melting curve (A’) 18S, (B’) *ACT*, (C’) *EF1*α**, (D’) *UBQ*, (E’) *RbcL*, (F’) ** β*-TUB* (G’) *GAPDH* and (H’) *NHX*

10.7717/peerj.5226/supp-2 Figure S2 Primer efficiency Primer efficiency based on standard graphs between target DNA dilutions vs. Cq values of seven reference genes and one target genes. (A) 18S, (B) *ACT*, (C) *EF1 α*, (D) *UBQ*, (E) *RbcL*, (F) ** β*-TUB* (G) *GAPDH* and (H) *NHX.*

10.7717/peerj.5226/supp-3 Figure S3 Average expression stability geNorm analysis of average expression stability value (M) for candidate reference genes shows most stable and least stable genes (A) Physiological tissue samples (B) in salt stress samples.

10.7717/peerj.5226/supp-4 Dataset S1 Dataset of Cq values Data for Cq value used for geNorm, NormFinder, BestKeeper, delta Ct analysis.

10.7717/peerj.5226/supp-5 Dataset S2 Bestkeeper analysis BestKeeper analysis for candidate reference genes and correlation coefficient (*r*) analysis performed for physiological tissue samples such as leaf, shoot, root, and flower.

10.7717/peerj.5226/supp-6 Dataset S3 BestKeeper analysis BestKeeper analysis for candidate reference genes and correlation coefficient (*r*) analysis performed for salt stress leaf samples.

## Additional Information and Declarations

### Data Availability

The following information was supplied regarding data availability: The raw data are provided as Supplemental Files.
