# Supplementary figures and images for: Selection of reference genes for quantitative real-time PCR analysis in halophytic plant Rhizophora apiculata
Source: PeerJ. 2018 Jul 12;6:e5226. doi: 10.7717/peerj.5226 (PMC6046198; doi:10.7717/peerj.5226)

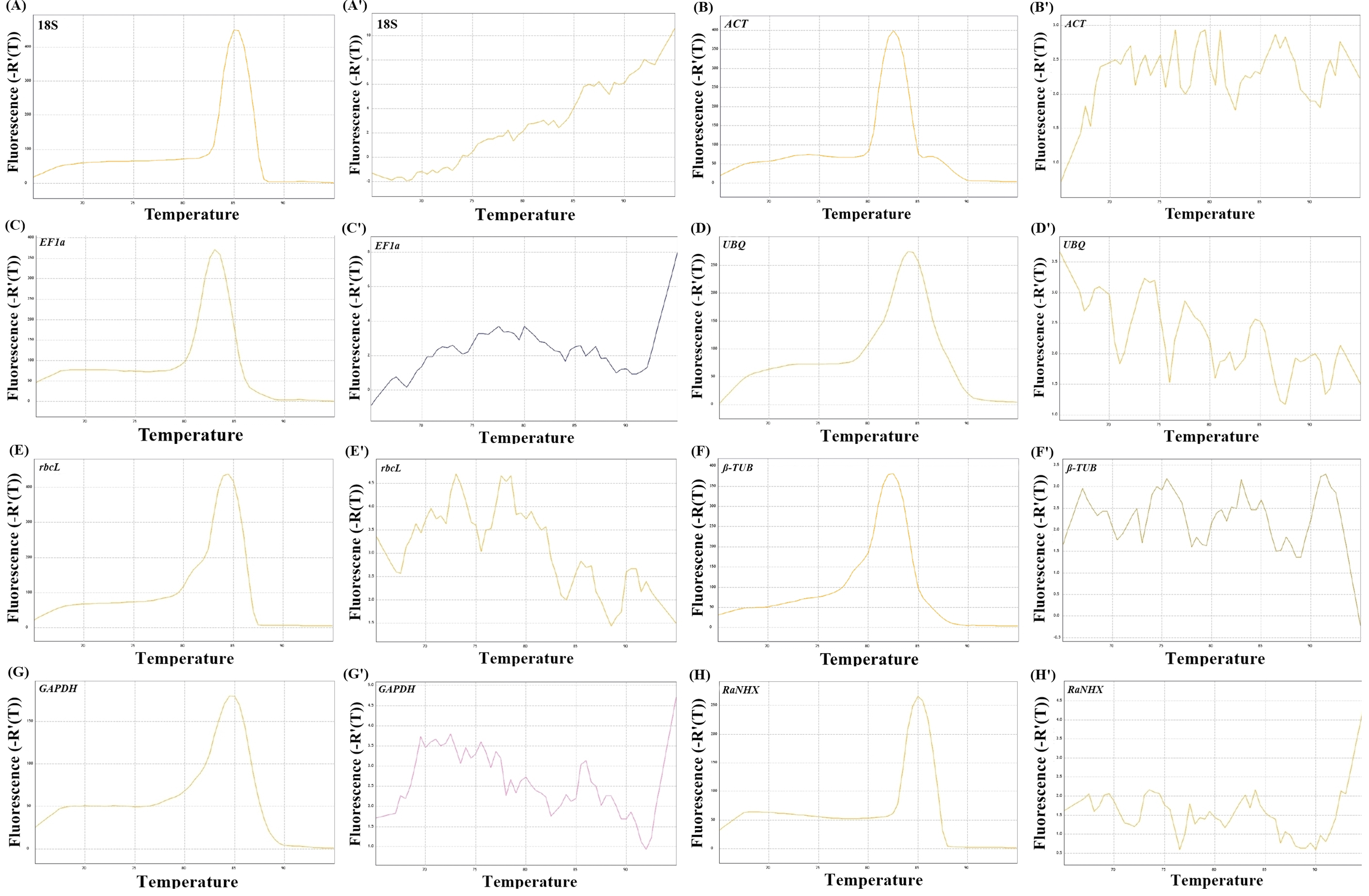

Supplement: Figure S1 — qRT-PCR template and negative control (NTC) melting curve. Template melting curve (A) 18S, (B) ACT, (C) EF1α, (D) UBQ, (E) RbcL, (F) β-TUB (G) GAPDH and (H) NHX. Negative control samples without template (NTC) melting curve (A’) 18S, (B’) ACT, (C’) EF1α, (D’) UBQ, (E’) RbcL, (F’) β-TUB (G’) GAPDH and (H’) NHX [file peerj-06-5226-s001.png]

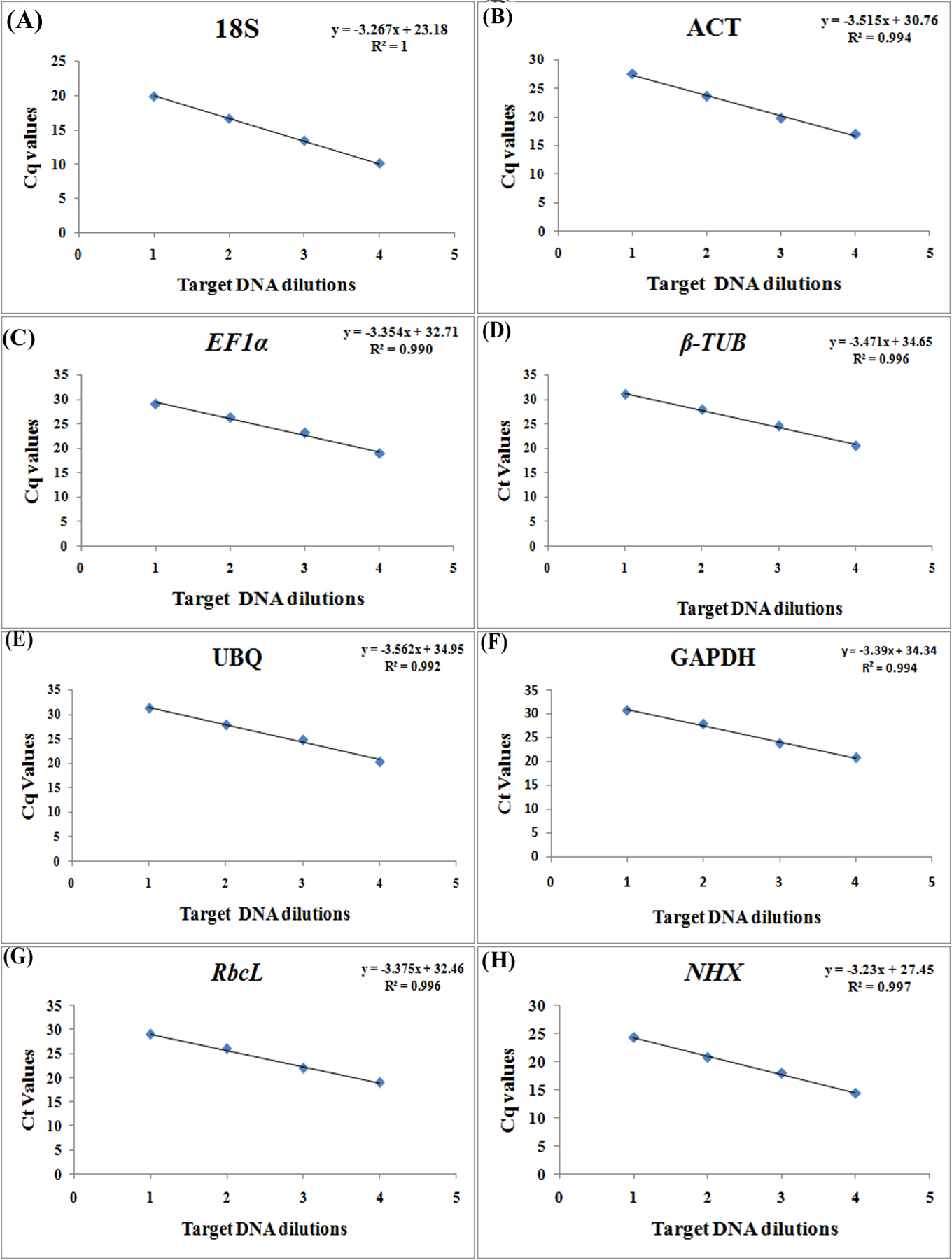

Supplement: Figure S2 — Primer efficiency based on standard graphs between target DNA dilutions vs. Cq values of seven reference genes and one target genes. (A) 18S, (B) ACT, (C) EF1 α, (D) UBQ, (E) RbcL, (F) β-TUB (G) GAPDH and (H) NHX. [file peerj-06-5226-s002.png]

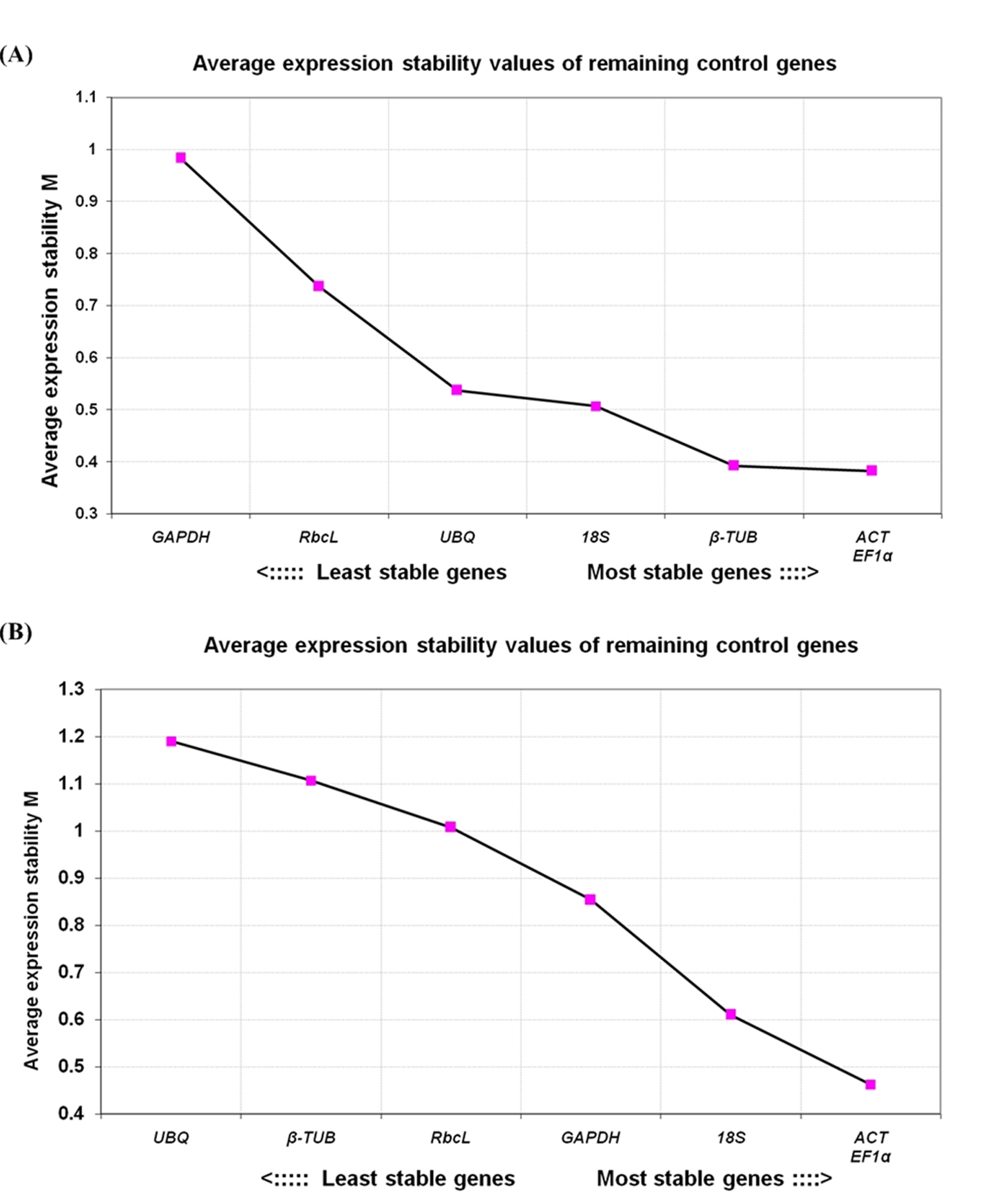

Supplement: Figure S3 — geNorm analysis of average expression stability value (M) for candidate reference genes shows most stable and least stable genes (A) Physiological tissue samples (B) in salt stress samples. [file peerj-06-5226-s003.png]
