# Supplementary material for: Selection of reference genes for quantitative real-time PCR analysis in halophytic plant Rhizophora apiculata
Source: PeerJ. 2018 Jul 12;6:e5226. doi: 10.7717/peerj.5226 (PMC6046198; doi:10.7717/peerj.5226)
Supplement: Dataset S1 — Data for Cq value used for geNorm, NormFinder, BestKeeper, delta Ct analysis. [file peerj-06-5226-s004.docx]

**Dataset Tables**

Cq values of seven candidate reference genes of *Rhizophora apiculata* in physiological conditions and salt stress tissue samples

**(A)**

|  | Reference Gene | Min | Max | Average | SD | SE | 1^st^ Quartile | 3^rd^ Quartile | Median |
| --- | --- | --- | --- | --- | --- | --- | --- | --- | --- |
| Physiolo-gical Tissue samples Cq values | *18S* | 12.27 | 15.07 | 14.16 | 0.831 | 0.26 | 13.78 | 14.8 | 14.38 |
|  | *EF1α* | 18 | 22.08 | 19.986 | 1.21 | 0.38 | 19.12 | 20.72 | 20.27 |
|  | *ACTIN* | 19.3 | 22.61 | 21.115 | 1.29 | 0.4 | 19.94 | 22.14 | 21.69 |
|  | *β-TUB* | 19.06 | 22.15 | 20.619 | 1.19 | 0.37 | 19.72 | 21.59 | 20.59 |
|  | *GAPDH* | 19.83 | 27.36 | 21.771 | 2.226 | 0.704 | 20.645 | 21.88 | 21.13 |
|  | *UBQ* | 19.8 | 21.42 | 20.683 | 0.6421 | 0.20 | 20.01 | 21.25 | 20.95 |
|  | *rbcL* | 16.28 | 21.75 | 19.73 | 2.02 | 0.64 | 17.94 | 21.64 | 20.31 |
| Salt stress tissue samples Cq values | *18S* | 13.45 | 16.71 | 14.82 | 1.08 | 0.360 | 14.06 | 15.66 | 14.69 |
|  | *EF1 α* | 17.68 | 21.52 | 20.01 | 1.09 | 0.363 | 19.61 | 23.25 | 20.02 |
|  | *ACTIN* | 20.3 | 24.21 | 22.49 | 1.302 | 0.43 | 22.51 | 23.25 | 22.61 |
|  | *β-TUB* | 16.28 | 23.03 | 20.84 | 2.072 | 0.690 | 20.13 | 21.96 | 21.65 |
|  | *GAPDH* | 18.99 | 24.35 | 21.65 | 1.76 | 0.588 | 20.13 | 22.81 | 21.46 |
|  | *UBQ* | 20.01 | 25.24 | 23.27 | 1.93 | 0.64 | 22.47 | 24.54 | 23.98 |
|  | *rbcL* | 12.18 | 21.75 | 15.75 | 3.543 | 1.18 | 13.35 | 16.02 | 14.39 |

**(B)** Average Cq values and standard error’s of four reference gene used for sodium/proton antiporter gene (NHX)

|  | ***EF1α*** | | ***ACT*** | | **18S** | | ***UBQ*** | | ***EF1α+ ACT*** | |
| --- | --- | --- | --- | --- | --- | --- | --- | --- | --- | --- |
|  | Average | SE | Average | SE | Average | SE | Average | SE | Average | SE |
| 0 h | 1.037934 | 0.394 | 0.701008 | 0.0412 | 1.026588 | 0.24048 | 0.892191 | 0.0216404 | 1.114235 | 0.39444 |
| 6 h | 0.821119 | 0.2417 | 0.913853 | 0.006334 | 0.334914 | 0.10551 | 8.895805 | 1.59761 | 0.986148 | 0.24173 |
| 12 h | 6.299345 | 0.8012 | 2.118233 | 0.36666 | 4.959307 | 0.07474 | 5.797474 | 0.11481 | 4.371138 | 0.8012 |
| 24 hr | 1.381144 | 0.8382 | 0.902 | 0.11195 | 0.876795 | 0.02319 | 0.719293 | 0.2579 | 2.12107 | 0.838243 |

**(C) geNorm Analysis**: Physiological tissue samples lowest M value show the most stable reference genes and highest M value represents least stable genes


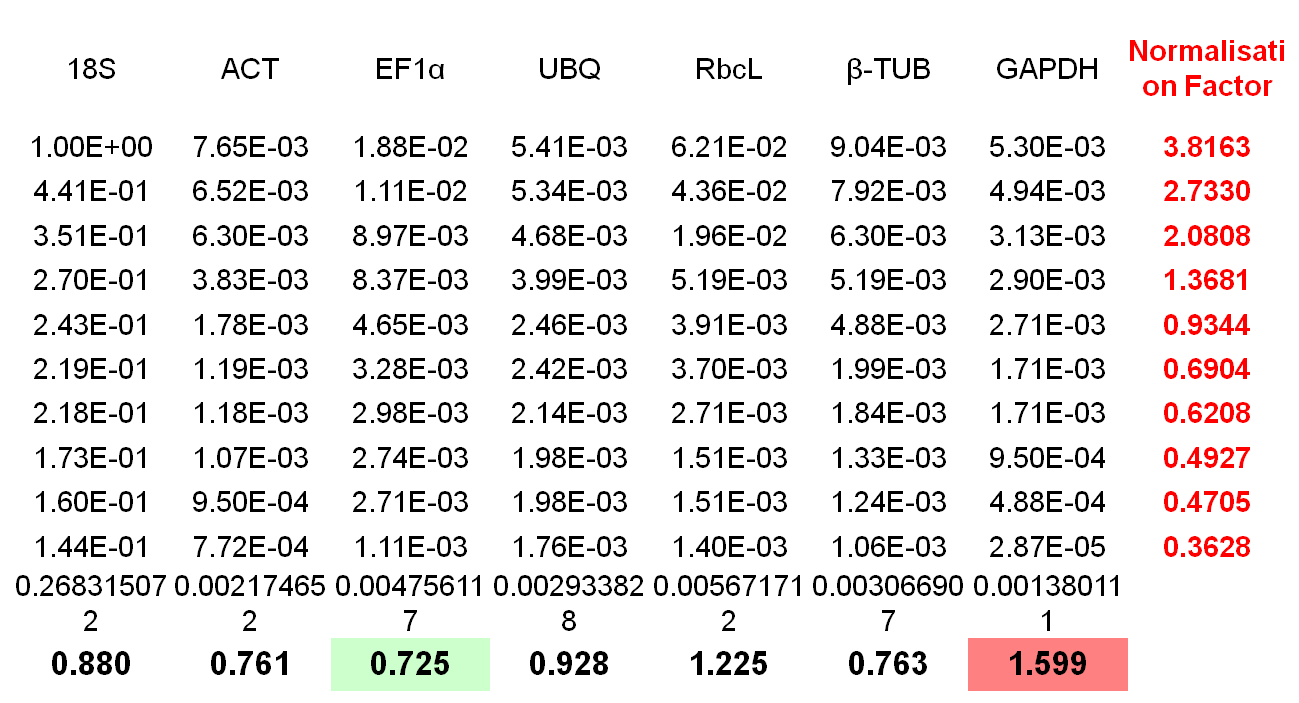


**M <1.5**

**(D) geNorm analysis:** In physiological tissue samples the most stable pair of gene

| **ACT** | **EF1α** | **Normalisation Factor** |
| --- | --- | --- |
| 7.65E-03 | 1.88E-02 | **3.7334** |
| 6.52E-03 | 1.11E-02 | **2.6491** |
| 6.30E-03 | 8.97E-03 | **2.3384** |
| 3.83E-03 | 8.37E-03 | **1.7599** |
| 1.78E-03 | 4.65E-03 | **0.8953** |
| 1.19E-03 | 3.28E-03 | **0.6158** |
| 1.18E-03 | 2.98E-03 | **0.5826** |
| 1.07E-03 | 2.74E-03 | **0.5324** |
| 9.50E-04 | 2.71E-03 | **0.4984** |
| 7.72E-04 | 1.11E-03 | **0.2883** |
| 0.002174652 | 0.004756117 |  |
| **0.382** | **0.382** |  |

**(E) geNorm Analysis**: salt stress samples lowest M value show the most stable reference genes and highest M value represents least stable genes


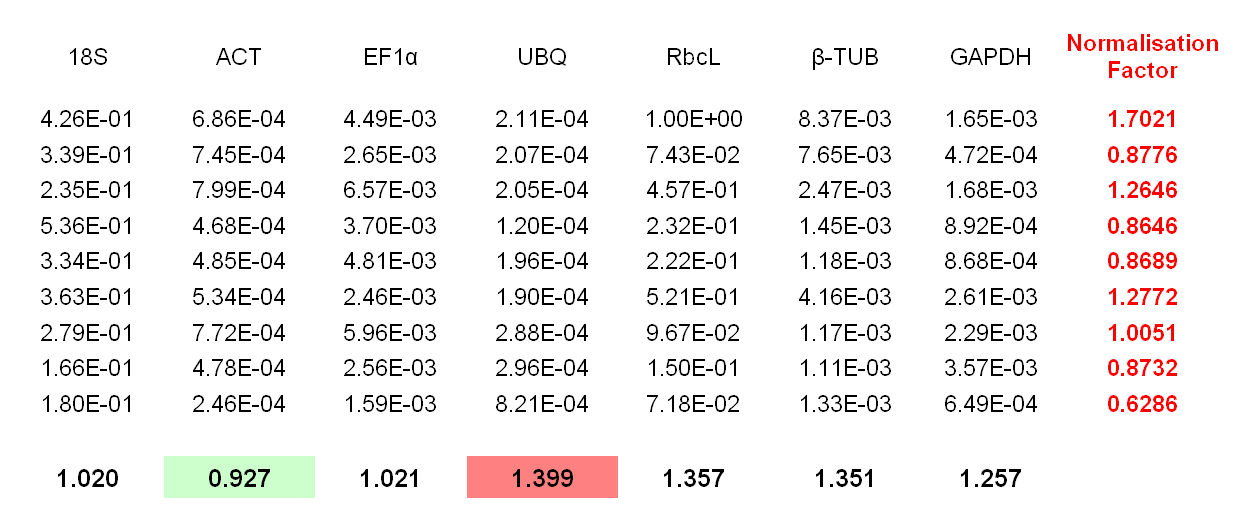


**M<1.5**

(F) geNorm Analysis: In salt stress samples the most stable pair of gene

| Actin | EF1α | Normalisation Factor |
| --- | --- | --- |
| 6.86E-04 | 4.49E-03 | **1.2628** |
| 7.45E-04 | 2.65E-03 | **1.0116** |
| 7.99E-04 | 6.57E-03 | **1.6491** |
| 4.68E-04 | 3.70E-03 | **0.9472** |
| 4.85E-04 | 4.81E-03 | **1.0994** |
| 5.34E-04 | 2.46E-03 | **0.8245** |
| 7.72E-04 | 5.96E-03 | **1.5440** |
| 4.78E-04 | 2.56E-03 | **0.7965** |
| 2.46E-04 | 1.59E-03 | **0.4496** |
| 0.0005485 | 0.003517799 |  |
| **0.462** | **0.462** |  |

**(G) NormFinder analysis**: physiological tissue sample lowest stability value show the most stable reference genes and highest value represents least stable genes

| **Gene name** | **Stability value** |
| --- | --- |
| 18S | 0.410 |
| ACT | 0.164 |
| EF1α | 0.085 |
| UBQ | 0.463 |
| RbcL | 0.500 |
| β-TUB | 0.135 |
| GAPDH | 0.568 |
|  |  |
| **Best gene** | EF1α |
| **Stability value** | 0.085 |
|  |  |
| **Best combination of two genes** | EF1α and β-TUB |
| **Stability value for best combination of two genes** | 0.070 |

**(H) NormFinder analysis:** Salt stress samples lowest stability value show the most stable reference genes and highest value represents least stable genes

|  |  |
| --- | --- |
| **Gene name** | **Stability value** |
| 18S | 0.273 |
| ACT | 0.196 |
| EF1α | 0.257 |
| UBQ | 0.518 |
| RbcL | 0.499 |
| β-TUB | 0.533 |
| GAPDH | 0.483 |
|  |  |
| **Best gene** | **ACT** |
| **Stability value** | **0.196** |
|  |  |
| **Best combination of two genes** | ACT and EF1α |
| **Stability value for best combination of two genes** | 0.183 |
