# Supplementary material for: Selection of reference genes for quantitative real-time PCR analysis in halophytic plant Rhizophora apiculata
Source: PeerJ. 2018 Jul 12;6:e5226. doi: 10.7717/peerj.5226 (PMC6046198; doi:10.7717/peerj.5226)
Supplement: Dataset S2 — BestKeeper analysis for candidate reference genes and correlation coefficient (r) analysis performed for physiological tissue samples such as leaf, shoot, root, and flower. [file peerj-06-5226-s005.docx]

| CP data of housekeeping Genes by BEST KEEPER | | | | | | | |
| --- | --- | --- | --- | --- | --- | --- | --- |
|  | 18S | ACT | EF1α | UBQ | RbcL | β-TUB | GAPDH |
| geo Mean [CP] | 14.14 | 21.08 | 19.95 | 20.67 | 19.63 | 20.59 | 21.68 |
| AR Mean [CP] | 14.17 | 21.12 | 19.99 | 20.68 | 19.73 | 20.62 | 21.77 |
| min [CP] | 12.27 | 19.30 | 18.00 | 19.80 | 16.28 | 19.06 | 19.83 |
| max [CP] | 15.07 | 22.61 | 22.08 | 21.42 | 21.75 | 22.15 | 27.36 |
| std dev [+/- CP] | 0.60 | 1.15 | 0.99 | 0.57 | 1.64 | 1.08 | 1.53 |
| CV [% CP] | 4.25 | 5.45 | 4.95 | 2.77 | 8.30 | 5.23 | 7.01 |
| min [x-fold] | -3.67 | -3.43 | -3.87 | -1.83 | -10.22 | -2.88 | -3.60 |
| max [x-fold] | 1.90 | 2.89 | 4.37 | 1.68 | 4.34 | 2.95 | 51.34 |
| std dev [+/- x-fold] | 1.52 | 2.22 | 1.98 | 1.49 | 3.11 | 2.11 | 2.88 |

| Pearson correlation coefficient ( r ) by BEST KEEPER | | | | | | | | | | | | | |
| --- | --- | --- | --- | --- | --- | --- | --- | --- | --- | --- | --- | --- | --- |
|  | 18S | | ACT | | EF1α | | UBQ | | RbcL | | β-TUB | | GAPDH |
| ACT | 0.888 | | - | | - | | - | | - | | - | | - |
| p-value | 0.001 | | - | | - | | - | | - | | - | | - |
| EF1α | 0.917 | | 0.956 | | - | | - | | - | | - | | - |
| p-value | 0.001 | | 0.001 | | - | | - | | - | | - | | - |
| UBQ | 0.886 | | 0.992 | | 0.952 | | - | | - | | - | | - |
| p-value | 0.001 | | 0.001 | | 0.001 | | - | | - | | - | | - |
| RbcL | 0.948 | | 0.949 | | 0.916 | | 0.955 | | - | | - | | - |
| p-value | 0.001 | | 0.001 | | 0.001 | | 0.001 | | - | | - | | - |
| β-TUB | 0.869 | | 0.953 | | 0.945 | | 0.939 | | 0.912 | | - | | - |
| p-value | 0.001 | | 0.001 | | 0.001 | | 0.001 | | 0.001 | | - | | - |
| GAPDH | 0.699 | | 0.731 | | 0.860 | | 0.728 | | 0.695 | | 0.793 | | - |
| p-value | 0.025 | | 0.016 | | 0.001 | | 0.017 | | 0.026 | | 0.006 | | - |
| Pearson correlation coefficient ( r ) | | | | | | | | | | | | | |
| BestKeeper vs. | | 18S | | ACT | | EF1α | | UBQ | | RbcL | | β-TUB | GAPDH |
| coeff. of corr. [r] | | 0.935 | | 0.966 | | 0.987 | | 0.964 | | 0.958 | | 0.964 | 0.850 |
| p-value | | 0.001 | | 0.001 | | 0.001 | | 0.001 | | 0.001 | | 0.001 | 0.002 |
