# Supplementary material for: Selection of reference genes for quantitative real-time PCR analysis in halophytic plant Rhizophora apiculata
Source: PeerJ. 2018 Jul 12;6:e5226. doi: 10.7717/peerj.5226 (PMC6046198; doi:10.7717/peerj.5226)
Supplement: Dataset S3 — BestKeeper analysis for candidate reference genes and correlation coefficient (r) analysis performed for salt stress leaf samples. [file peerj-06-5226-s006.docx]

| CP data of housekeeping Genes by BEST KEEPER | | | | | | | |
| --- | --- | --- | --- | --- | --- | --- | --- |
|  | 18S | *ACT* | *EF1α* | *UBQ* | *RbcL* | *Β-TUB* | *GAPDH* |
| geo Mean [CP] | 14.79 | 22.46 | 19.99 | 23.2 | 15.44 | 20.74 | 21.59 |
| AR Mean [CP] | 14.82 | 22.49 | 20.01 | 23.27 | 15.75 | 20.84 | 21.65 |
| min [CP] | 13.45 | 20.3 | 17.68 | 20.01 | 12.18 | 16.28 | 18.99 |
| max [CP] | 16.71 | 24.21 | 21.52 | 25.24 | 21.75 | 23.03 | 24.35 |
| std dev [+/- CP] | 0.85 | 0.93 | 0.75 | 1.58 | 2.7 | 1.53 | 1.38 |
| CV [% CP] | 5.77 | 4.15 | 3.75 | 6.78 | 17.14 | 7.32 | 6.38 |
| min [x-fold] | -2.53 | -4.46 | -4.95 | -9.12 | -9.56 | -22.04 | -6.05 |
| max [x-fold] | 3.78 | 3.37 | 2.89 | 4.12 | 79.52 | 4.88 | 6.79 |
| std dev [+/- x-fold] | 1.81 | 1.91 | 1.68 | 2.99 | 6.5 | 2.88 | 2.6 |

| Pearson correlation coefficient ( r ) by BEST KEEPER | | | | | | | |
| --- | --- | --- | --- | --- | --- | --- | --- |
|  | 18S | *ACT* | *EF1α* | *UBQ* | *RbcL* | *Β-TUB* | *GAPDH* |
| Actin | 0.21 | - | - | - | - | - | - |
| p-value | 0.587 | - | - | - | - | - | - |
| eEF | -0.318 | 0.713 | - | - | - | - | - |
| p-value | 0.405 | 0.031 | - | - | - | - | - |
| Ubq | 0.717 | 0.263 | -0.398 | - | - | - | - |
| p-value | 0.03 | 0.494 | 0.289 | - | - | - | - |
| RbcL | -0.557 | -0.258 | 0.417 | -0.937 | - | - | - |
| p-value | 0.119 | 0.502 | 0.264 | 0.001 | - | - | - |
| Tubulin | 0.489 | 0.797 | 0.236 | 0.688 | -0.725 | - | - |
| p-value | 0.182 | 0.01 | 0.54 | 0.041 | 0.027 | - | - |
| GAPDH | 0.215 | 0.275 | 0.323 | -0.152 | 0.361 | -0.145 | - |
| p-value | 0.579 | 0.474 | 0.396 | 0.696 | 0.34 | 0.709 | - |

| Pearson correlation coefficient ( r ) | | | | | | | |
| --- | --- | --- | --- | --- | --- | --- | --- |
| BestKeeper vs. | 18S | *ACT* | *EF1α* | *UBQ* | *RbcL* | *Β-TUB* | *GAPDH* |
| coeff. of corr. [r] | 0.349 | 0.769 | 0.71 | 0.008 | 0.17 | 0.411 | 0.714 |
| p-value | 0.358 | 0.015 | 0.032 | 0.983 | 0.661 | 0.272 | 0.031 |
